# A systematic review of associations between gut microbiota composition and growth failure in preterm neonates

**DOI:** 10.1080/19490976.2023.2190301

**Published:** 2023-03-16

**Authors:** Larissa L. Neves, Amy B. Hair, Geoffrey A. Preidis

**Affiliations:** a Division of Gastroenterology, Hepatology, & Nutrition, Department of Pediatrics, Baylor College of Medicine and Texas Children’s Hospital, Houston, TX, USA; b Division of Neonatology, Department of Pediatrics, Baylor College of Medicine and Texas Children’s Hospital, Houston, TX, USA

**Keywords:** Growth failure, gut microbiome, malnutrition, neonate, postnatal growth, preterm infant, underweight

## Abstract

Growth failure is among the most prevalent and devastating consequences of prematurity. Up to half of all extremely preterm neonates struggle to grow despite modern nutrition practices. Although elegant preclinical models suggest causal roles for the gut microbiome, these insights have not yet translated into biomarkers that identify at-risk neonates or therapies that prevent or treat growth failure. This systematic review aims to identify features of the neonatal gut microbiota that are positively or negatively associated with early postnatal growth. We identified 860 articles, of which 14 were eligible for inclusion. No two studies used the same definitions of growth, ages at stool collection, and statistical methods linking microbiota to metadata. In all, 58 different taxa were associated with growth, with little consensus among studies. Two or more studies reported positive associations with Enterobacteriaceae, *Bacteroides*, *Bifidobacterium*, *Enterococcus*, and *Veillonella*, and negative associations with *Citrobacter, Klebsiella*, and *Staphylococcus*. *Streptococcus* was positively associated with growth in five studies and negatively associated with growth in three studies. To gain insight into how the various definitions of growth could impact results, we performed an exploratory secondary analysis of 245 longitudinally sampled preterm infant stools, linking microbiota composition to multiple clinically relevant definitions of neonatal growth. Within this cohort, every definition of growth was associated with a different combination of microbiota features. Together, these results suggest that the lack of consensus in defining neonatal growth may limit our capacity to detect consistent, meaningful clinical associations that could be leveraged into improved care for preterm neonates.

## Introduction

More than 50,000 premature, very low birth weight infants are born in the US each year. Half of these neonates develop malnutrition and postnatal growth failure, the etiology of which is largely unknown. Numerous studies report intriguing associations between the preterm infant gut microbiota and neonatal growth; however, these findings have not yet translated into clinically impactful therapies.

A causal link between the gut microbiome and early postnatal growth failure was first identified in studies of Bangladeshi^1^ and Malawian^2,3^ infants and children. Malnourished children with kwashiorkor have an “immature” gut microbiome, which is characterized by delayed acquisition of microbial functions^3^ and age-discriminatory bacterial species.^1^ Gnotobiotic mouse recipients of these immature microbiota have poor growth compared to mice receiving microbiota from healthy children;^3^ growth failure can be prevented by colonization with age- and growth-discriminatory microbes.^2^ Interestingly, the growth-promoting potential of microbiota in weanling germ-free mice depends on the developmental stage of the human donor. Mice receiving microbiota from 6-month-old infants grow more compared to those receiving microbes from 18-month-old toddlers,^2^ whereas mice receiving meconium microbiota from full-term newborns grow more compared to recipients of meconium microbiota from very preterm newborns.^4^

**Figure 1.**
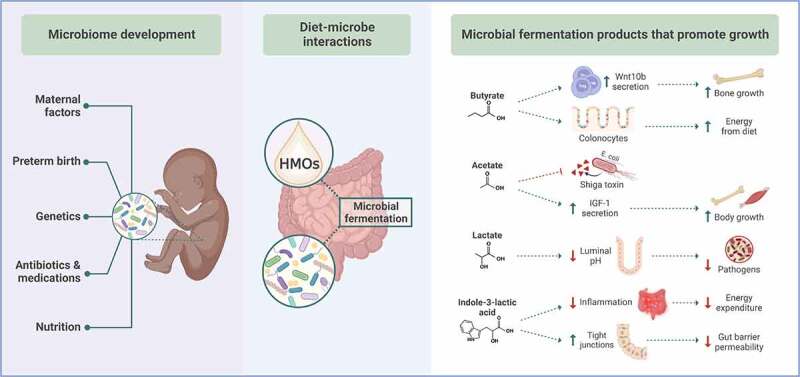
Mechanisms by which microbial fermentation of human milk oligosaccharides promote neonatal growth. Numerous factors shape the development of the neonatal gut microbiome. One of the most important factors – nutrition – provides substrates for gut microbes to perform beneficial functions that stimulate neonatal growth. Non-digestible HMOs are metabolized by bacterial enzymes to produce bioavailable energy, growth-promoting metabolites, and anti-inflammatory factors. HMO: human milk oligosaccharides; IGF-1: insulin-like growth factor-1; Wnt10b: Wnt family member 10b. Figure created with BioRender.Com.

Preclinical models highlight mechanisms that may underlie these causal links (Figure 1). Microbes ferment non-digestible dietary substrates into absorbable energy, including short-chain fatty acids (SCFAs) that enhance postnatal growth.^5–8^ Butyrate fed to germ-free mice or produced in the mouse intestine by *Lacticaseibacillus rhamnosus* GG promotes bone growth by inducing regulatory T cells to secrete the bone-anabolic Wnt ligand Wnt10b,^8^ whereas acetate produced by specific strains of *Bifidobacterium* protects mice from death by enterohemorrhagic *Escherichia coli* in part by blocking translocation of Shiga toxin from the intestine into the bloodstream.^9^ Notably, elevated levels of fecal butyrate and propionate are observed among extremely premature infants with severe brain injury;^10^ however, SCFA levels can be affected by numerous physiologic changes including altered absorption and colonocyte utilization.

For the neonate, non-digestible dietary substrates include human milk oligosaccharides (HMOs). Based on the finding that HMOs are deficient in breast milk from mothers of malnourished infants in Malawi, milk oligosaccharides were fed to mice or piglets, and this resulted in increased weight gain, bone mineral density, and cortical thickness – but only if gut bacteria were present.^11^ Milk oligosaccharides reduce osteoclasts and the bone resorption marker CTX-1 in mice in a microbiota-dependent manner, suggesting that microbial metabolism of HMOs promotes postnatal weight gain in part by inhibiting osteoclastogenesis and bone resorption.^12^ HMO fermentation produces other factors that may be growth-promoting, including the anti-inflammatory metabolite indole-3 lactic acid,^13^ and might reduce the risk of bacterial translocation via increased expression of colonocyte tight junction proteins.^14^ Finally, gut microbes influence the somatotropic axis to promote growth. *Drosophila* larvae monocolonized by bacteria that are unable to produce acetate exhibit poor growth and altered insulin/insulin-like growth factor (IGF) signaling; acetate supplementation rescues these deficits.^6^ Similarly, germ-free mice have reduced levels of IGF-1 and stunted growth compared to conventional mice. Colonizing germ-free mice with microbial communities^15^ or with strains of growth-promoting lactobacilli^16^ restores somatotropic axis activity, bone growth, and weight gain. These results indicate that in well-defined laboratory conditions, gut microbes and their metabolites regulate postnatal growth.

These intriguing preclinical findings instill hope that the newborn gut microbiome could be leveraged in the neonatal intensive care unit, both as a source of biomarkers that identify infants at risk of growth failure and as a growth-promoting therapeutic target. Thus, the purpose of this systematic review is to examine the data associating specific features of the human preterm infant gut microbiome to neonatal growth. Identifying these relationships could open the door for new microbiota-targeted therapies that prevent or treat neonatal growth failure and its short- and long-term consequences.

## Results

### Characteristics of articles identified by systematic review

**Figure 2.**
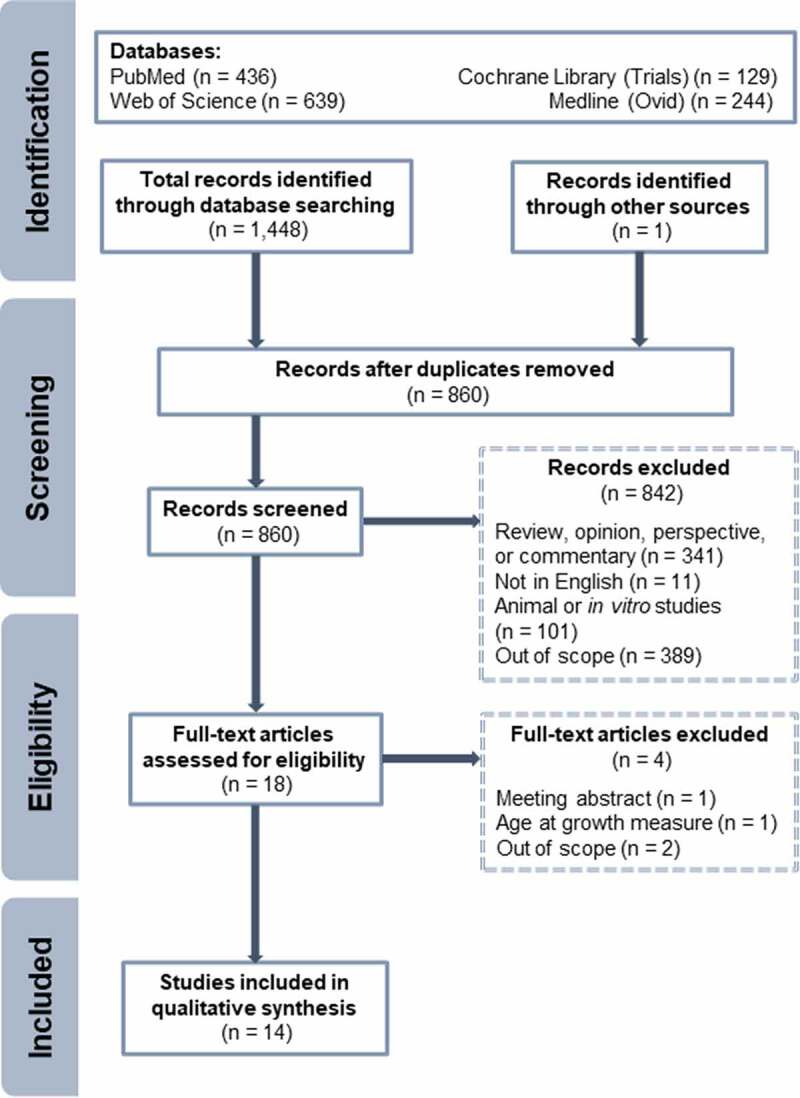
Preferred Reporting Items for Systematic Reviews and Meta-Analyses flow diagram of studies identified and included.

Our literature search identified 860 records, of which 14 met criteria to be included in our qualitative synthesis of studies that report associations between human gut microbial community composition and neonatal growth (Figure 2). Study characteristics are presented in Table 1. Two studies were randomized controlled trials (one tested enhanced vs standard parenteral nutrition;^17^ one tested probiotic *Limosilactobacillus reuteri* DSM 17938 vs placebo^18^). Of the 12 observational studies, 10 were prospective cohort studies,^19–28^ one was a nested case-control study,^29^ and one was a retrospective cohort study.^30^ There was geographic diversity, with five studies conducted in China,^23,26–29^ four each in Europe^17–19,25^ and the United States,^20–22,30^ and one in Brazil.^24^ Some studies included only extremely preterm infants (< 28 weeks gestation), whereas others included any preterm infant (< 37 weeks), resulting in a range of extremely low birth weight (< 1000 g) to low birth weight (< 2500 g). Newborn diets varied in parenteral and enteral nutrition (mother’s milk, pasteurized donor milk, fortifier, and formula). In two studies, microbiota analyses were limited to a single sample per patient,^23,24^ but in the remainder of studies stool was collected longitudinally. One study determined the abundance of select bacterial taxa using quantitative polymerase chain reaction;^19^ the other 13 studies sequenced subunits of the 16S rRNA gene. None of the studies employed whole metagenomic sequencing.

**Table 1.** Characteristics of studies identified by the systematic review and included in the qualitative synthesis.

Author & year	Geographic location	Study type	n =	GA at birth	Birth weight	Neonatal diet	Sample collection	Sequencing strategy
Arboleya et al 2017	Northern Spain	Prospective cohort	63	28–33	1085 g − 1580 g	Mixed feeding of breast milk and infant formula	Fecal samples on day of life 2, 10, and 30 (*n* = ~189)	qPCR of 8 taxa
Grier et al 2017	Rochester, NY, USA	Prospective cohort	95	28.83 (mean)	1290 g (mean)	− Breast milk or premature formula milk − Fortifiers, parenteral and enteral feeding	Meconium and fecal rectal swabs weekly from 24 to 46 weeks PMA (*n* = 719)	16S rRNA V3-V4
Younge et al 2019	Durham, NC, USA	Prospective cohort	58	26 (median)	800 g (median)	− Human milk given as initial diet − Parenteral and enteral feeding	Fecal samples weekly for 9 weeks upon reaching full enteral feeding (*n* = 385)	16S rRNA V4
Yee et al 2019	Tampa, FL, USA	Prospective cohort	83	24–37	< 1400 g	− Mother’s milk, donor milk, and formula (> 93% breastfed and > 64% exclusively breastfed) − Fortifiers and enteral feeding	Fecal samples at 1–6 weeks, and at 2 and 4 years of age including mother’s (*n* = 425)	16S rRNA V4
Li et al 2019	Shenzhen, China	Nested case-control	23	< 32	< 1500 g	Exclusively breastfed (> 21%), exclusively formula fed (> 34%), or mixed breast milk and formula feeding (> 43%)	Swab of meconium and fecal samples at 1 and 28 days of life (*n* = 44)	16S rRNA V3-V4
Blakstad et al 2019	Oslo, Norway	Randomized controlled trial (Enhanced parenteral nutrition intervention)	45	< 28	< 1500 g	− Mother’s milk or donor milk (not pasteurized) − Fortifiers, parenteral and enteral feeding	Fecal samples every 1–2 weeks from birth to hospital discharge (*n* = 264)	16S rRNA V1-V3
Zhang et al 2019	Shanghai, China	Prospective cohort	59	< 30	< 1200 g	− Exclusively human milk fed (> 74%), exclusively formula fed (> 10%), human milk mixed with formula (> 6%), or human milk mixed with thickener (> 8%) − Thickener was starch- or carbohydrate-based	One fecal sample at discharge (*n* = 59)	16S rRNA V3-V4
Terrazzan et al 2020	Porto Alegre, Brazil	Prospective cohort	63	< 33	1375 g (mean)	Exclusively breastfed (> 14%), exclusively formula fed (> 28%), or breast milk mixed with formula (> 57%)	First meconium sample (*n* = 63)	16S rRNA V4
Marti et al 2021	Stockholm/Linkoping, Sweden	Randomized, double-blind, placebo-controlled trial (*L.reuteri* DSM 17938)	132	23 - < 28	< 1000 g	− Exclusively mother’s milk or donor milk fed until weight reached 2000 g − Fortifiers, parenteral and enteral feeding	Fecal samples at 1–4 weeks of life, 36 weeks PMA, and 2 years of age (*n* = 558)	16S rRNA V3-V4
Heida et al 2021	Groningen, Netherlands	Prospective cohort	41	26–30	430–990 g	Mother’s milk, formula, or mixed	Meconium/fecal samples weekly during first 4 weeks of life (*n* = 142)	16S rRNA V3-V4
Ding et al 2021	Beijing, China	Prospective cohort	22	< 32	Not specified	− Breast milk and/or formula − Parenteral feeding when total enteral feeding not achieved	Fecal samples at 14 and 28 days of life (*n* = 44)	16S rRNA (V region not specified)
Shen et al 2022	Guangzhou, China	Prospective cohort	118	< 35	< 2000 g	Not specified	Fecal samples 1–2 times weekly from birth until hospital discharge (*n* = 467)	16S rRNA V4
Tadros et al 2022	Tampa, FL, USA	Retrospective	34	< 33	< 1500 g	− Exclusively breastfed (25%), exclusively formula fed (51%), or mixed feeding (24%)	Fecal samples weekly from birth to 28 days of life, additional sample at 36 weeks CGA (*n* = not specified)	16S rRNA V4
Huang et al 2022	Shenzhen, China	Prospective cohort	67	< 37	< 2500 g	− Mother’s milk or donor breast milk − Parenteral and enteral feeding	Meconium/fecal samples at day of life 0–1, 3, 4, and weekly for 10 weeks (*n* = not specified)	16S rRNA V4

CGA: corrected gestational age; GA: gestational age; PMA: post-menstrual age; qPCR: quantitative polymerase chain reaction; rRNA: ribosomal ribonucleic acid.

### Gut microbiota associations with neonatal growth

Each study used different statistical methods to associate microbiota composition with postnatal growth (Table 2). In some cases, microbiota from fecal samples obtained within the first ten days of life were associated with anthropometric indices at a point in time weeks to months later,^19,24,29^ whereas other studies used various methods to link longitudinally sampled microbiota to rates of growth.^17,20,22^ Only one study identified associations between growth and absolute bacterial abundance based on quantitative polymerase chain reaction.^19^ Given this heterogeneity of statistical techniques, we sought to determine whether any microbiota features consistently associated with appropriate growth or growth failure – irrespective of how the associations were computed in the individual studies.

**Table 2.** Gut microbiota associations with postnatal growth.

Author & year	Methods used to determine growth associations	Results
Arboleya et al 2017	- Microbiota at day of life 2 and 10 associated with weight gained at day of life 30 by univariate and multivariate generalized linear model adjusted for confounders (BW and GA) on forest plot - Growth metric: Fenton	- *Staphylococcus* and *Enterococcus* negatively associated with weight gain - *Weissella* positively associated with weight gain - Enterobacteriaceae and *Streptococcus* at day of life 2 positively associated with weight gain at 30 days of life - *Bacteroides* at day of life 10 positively associated with weight gain at 30 days of life
Grier et al 2017	- Change in weight z-score from birth to hospital discharge associated with “phase” of microbiota using linear mixed-effect regression models - Growth metric: Fenton	Delayed transition to “phase 3” (denoted as “Clostridia”) negatively associated with change in weight z-score
Younge et al 2019	- Infants grouped into severe postnatal growth failure (defined as weight < 3rd percentile at 40 weeks PMA or hospital discharge) vs appropriate growth - Shannon diversity compared between groups using smoothing spline ANOVA - Taxon relative abundance compared between groups using fitTimeSeries - Beta diversity compared between groups using PCoA on Jensen-Shannon divergences - Catch-up growth (defined as a positive change in weight z-score) associated with relative abundance using zero-inflated Log-normal mixture model - Growth metric: Fenton	- Growth failure group associated with low alpha diversity - Increased abundance of *Staphylococcus* in early weeks of life associated with growth failure - Dominance of Enterobacteriaceae (genus level: *Citrobacter*, *Enterobacter*, *Serratia*, and *Klebsiella*) in later weeks of life associated with growth failure - Increased abundance of Veillonellaceae, Streptococcaceae, Peptostreptococcaceae, Micrococcaeae, Lachnospiraceae, and Bacillaceae associated with appropriate growth - Change in weight z-score positively associated with *Streptococcus*, *Bifidobacterium*, Clostridiaceae, Clostridiales, Lachnospiraceae, Peptostreptococcaceae, *Veillonella*, and increased alpha-diversity - Change in weight-z-score negatively associated with *Staphylococcus*
Yee et al 2019	- Shannon diversity correlated with weight gain velocity (g/week) via regression adjusted for PMA - Beta diversity using unweighted UniFrac PCoA and PERMANOVA associated with improved length (a binary variable defined as whether the infant had a length-for-age z-score that was better than expected during the hospital stay) - Regression model that predicts PMA in relation to microbiome composition used to generate associations with improved length - Relative abundance associated with weight gain from birth to hospital discharge using analysis of composition of microbiomes (ANCOM) - Growth metric: Fenton	- Weak correlation of increased alpha diversity with overall weight growth rate - Increased “beta diversity volatility” positively associated with length - Reduced microbiome maturity associated with improved length - *Klebsiella* and *Staphylococcus* ESVs negatively associated with weight gain
Li et al 2019	- Infants grouped into EUGR (defined as weight < 10th percentile at hospital discharge) vs AGA - Group comparisons using LEfSe to identify differences in relative abundance - Growth metric: Clark et al 2003	- Day 1 samples: decreased alpha diversity (Shannon index) in AGA group - Day 1 samples: increased abundance of *Parabacteroides*, *Ruminococcus*, *Blautia*, and *Aeromonas* negatively associated with growth (increased abundance in EUGR) - Day 1 samples: increased abundance of *Aeromicrobium* and *Serratia* positively associated with growth (decreased abundance in EUGR) - Day 28 samples: Increased abundance of *Bacteroides*, *Parabacteroides*, *Eubacterium*, *Granulicatella*, and *Eggerthella* negatively associated with growth (more abundant in EUGR) - Day 28 samples: Increased abundance of *Salinivibrio* positively associated with growth (less abundant in EUGR)
Blakstad et al 2019	- Infants grouped into positive or negative change in weight z-score from birth to 36 weeks PMA associated with microbiome features using repeated-measures linear mixed models adjusted for BW or BW z-score and other covariates (i.e. volume of milk intake on first day of life) - Growth metric: Fenton (non-sex-specific) and Sjaerven’s (sex-specific) growth charts	- Increased richness (OTUs) and increased relative abundance of *Bifidobacterium* positively associated with growth - When adjusted for milk intake, no significant differences in richness
Zhang et al 2019	- Infants grouped into EUGR (defined as weight < 10th percentile at hospital discharge) or non-EUGR - Differences in relative abundance using multivariate logistic regression models adjusted for potential confounders (i.e. BW, feeding status) and FDR - Growth metric: Clark et al 2003	- Abundance of *Rothia*, *Pantoea*, *Citrobacter*, and *Kluyvera* negatively associated with growth (increased in EUGR) - Abundance of *Streptococcus Parasanguinis*_FW21 and Bacterium RB5FF6 negatively associated with growth (increased in EUGR) - Abundance of *Acinetobacter* sp_V12012 and *Enterobacter* sp CCBAU 15567 positively associated with growth (decreased in EUGR) - Abundance of *Klebsiella* (*granulomatis* and *michiganensis*) and *Microbacterium*_sp_TSWCW12 negatively associated with growth (increased in EUGR)
Terrazzan et al 2020	- Infants grouped into SGA (defined as weight < 10th percentile) vs AGA - Differences in taxon abundances analyzed using DESEq2 adjusted for FDR - Infants later grouped into HC catch-up growth (defined as ≤ 0.67 z-score variation between two consecutive z-scores) by or after 6 months of age - Growth metric: Fenton	- Abundance of Proteobacteria, *Acidobacteria* GP1, *Prevotella*, and *Polynucleobacter* positively associated with growth (increased in infants with AGA at birth) - Abundance of *Escherichia fergusoni* and *Streptococcus dentisani* positively associated with growth (increased in infants AGA at discharge) - Abundance of *Prevotella copri*, *Roseburia inulinivorans*, *Staphylococcus* sp, *Staphylococcus capitis*, *Sutterella stercoricanis*, *Corynebacterium tuberculostearicum*, and Ruminococcaceae negatively associated with growth (increased in infants with SGA at hospital discharge) - Increased alpha diversity (Shannon index) negatively associated with growth (increased in HC catch-up after 6 months) - Abundance of Bacteroidetes, Proteobacteria, *Salmonella*, *Flavobacterium*, and *Burkholderia* positively associated with growth (increased in HC catch-up by 6 months) - Abundance of *Prevotella*, *Enhydrobacter*, *Brevundimonas*, *Bradyrhizobium*, and *Acinetobacter* negatively associated with growth (increased in HC catch-up after 6 months)
Marti et al 2021	- Microbial diversity and richness correlated with HC growth velocity (change in z-score) using simple linear regression - Microbiota communities correlated with weight or HC growth velocities using envfit demonstrated on NMDS - Growth metric: Niklasson’s growth chart	- Positive association between microbiome diversity at 1 week and richness at 2 weeks of life with HC growth at 4 weeks of life - Positive association of microbial composition in weeks 1 and 3 with head growth at 4 weeks of life and 36 weeks PMA and with weight gain at 2 weeks, 4 weeks of life, and 36 weeks PMA
Heida et al 2021	- Microbiota associated with weight at the time of sample collection using simple linear regression and mixed effects regression - Growth metric: Dutch growth curve	- Abundance of Enterobacteriaceae positively associated with weight at weeks 3 and 4 of life - Ratio of Enterobacteriaceae to *Staphylococcus* positively associated with weight
Ding et al 2021	- Infants grouped into AGA (control) or EUGR (defined as weight < 10th percentile to corresponding GA or weight loss > 2 SD from birth to 14 or 28 days) - Microbiome differences via LEfSe analysis at 14 and 28 days of life; further analyses compared abundances between groups - Growth metric: Fenton	- At week 2 after birth, Enterococcaceae and *Enterococcus* had an LDA score > 4 in AGA infants and both were more abundant in AGA vs EUGR - At week 2 after birth, Streptococcaceae and *Streptococcus* had an LDA score > 4 in EUGR infants and both were more abundant in EUGR vs AGA
Shen et al 2022	- Microbiome associated with weight gain based on the age (days of life) at which infant reached 2 kg or based on weekly weight gain rate using generalized linear mixed models; further analyses divided infants into high or low groups based on weekly weight gain rate falling < 6% (converted from daily weight gain being < 10 g/kg) - Growth metric: not specified	- Abundance of *Streptococcus* negatively associated with age to reach 2 kg weight - Lower alpha diversity (Shannon index) positively associated with growth (higher weekly weight gain rate) - Abundance of *Streptococcus* positively associated with growth (decreased in low weekly weight gain rate group)
Tadros et al 2022	- Microbiome associated with weight z-score, length z-score, and change in weight and length z-scores using early growth from birth to 4 months of life via regression-based kernel association tests - Growth metric: Fenton	In early growth (birth to 4 months): - Abundance of *Streptococcus*, *Veillonella*, and *Haemophilus* positively associated with length z-score (samples from < 14 days of life) - Abundance of *Proteus* negatively associated with length z-score (samples from 14–28 days of life) - Abundance of *Bacteroides* and *Haemophilus* positively associated with weight z-score (samples from < 14 days of life) - Abundance of *Gemella* negatively associated with weight z-score (samples from 12–28 days of life) - Abundance of Gammaproteobacteria negatively associated with weight z-score (samples from 36 weeks CGA)
Huang et al 2022	- Infants grouped into EUGR (defined as weight < 10th percentile at hospital discharge) or control - Microbiome differences between groups identified using LEfSe, multivariate associations with linear models (adjusted for mode of delivery), and Wilcoxon rank-sum tests. - Growth metric: Fenton	- Abundance of Proteobacteria (Enterobactericeae and Moraxellaceae) negatively associated with growth (increased in EUGR) - Abundance of *Streptococcus* negatively associated with growth (decreased in control) - Abundance of *Clostridium* and *Veillonella* positively associated with growth (delayed increase in EUGR compared to faster increase in control)

AGA: appropriate for gestational age; ANOVA: analysis of variance; BW: birth weight; CGA: corrected gestational age; DEseq2: differential gene sequence; ESV: exact sequence variant; EUGR: extrauterine growth restriction; FDR: false discovery rate; GA: gestational age; HC: head circumference; LDA: linear discriminant analysis; LEfSe: linear discriminant analysis effect size; NMDS: non-metric multidimensional scaling; OTU: operational taxonomic unit; PCoA: principal coordinate analysis; PERMANOVA: permutational multivariate analysis of variance; PMA: post-menstrual age; SD: standard deviation; SGA: small for gestational age; UniFrac: unique fraction metric; VLBW: very low birth weight.

Gut microbial community characteristics that correlated either positively or negatively with neonatal growth are listed in Table 2. The Shannon diversity index was the most commonly reported alpha-diversity metric. Interestingly, neonatal growth and Shannon diversity were positively correlated in three studies^18,21,22^ and negatively correlated in three studies.^24,26,29^ Some studies calculated “microbiota maturity” as an index of a neonate’s postnatal acquisition of age-discriminatory taxa (microbes whose proportional representation define gut microbiota assembly within healthy infants sampled in the same cohort). Microbiota maturity and neonatal growth were positively correlated in one study^20^ and negatively correlated in another study.^22^

**Figure 3.**
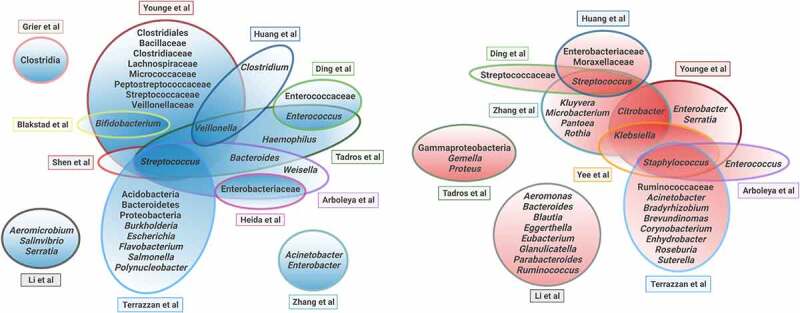
Microbial taxa positively or negatively associated with growth in studies of human preterm neonates. Blue Venn diagram (left) represents taxa positively associated with postnatal growth. Red Venn diagram (right) represents taxa negatively associated with postnatal growth. Figure created with BioRender.Com.

There was some agreement among the 14 studies correlating the relative abundance of specific taxa with growth (Figure 3). Five studies reported positive associations between *Streptococcus* and growth,^19,21,24,26,30^ whereas four studies reported negative associations between *Staphylococcus* and growth.^19,21,22,24^ Several taxa were positively correlated with growth in some studies and negatively correlated with growth in other studies; these included Enterobacteriaceae, Streptococcaceae, *Acinetobacter*, *Bacteroides*, *Enterobacter*, *Enterococcus*, *Serratia*, and *Streptococcus*. In one study, the relative abundance of *Prevotella* in the first meconium sample positively associated with appropriate weight at birth but negatively associated with growth at hospital discharge.^24^ In all, 58 distinct taxa were associated positively and/or negatively with growth. For the 32 taxa positively associated with growth, 26 (81%) were reported in a single study. For the 34 taxa negatively associated with growth, 30 (88%) were reported in a single study. Although all 14 studies identified specific microbiota features that were significantly associated with neonatal growth (Table 2), we found surprisingly little agreement among studies.

### Variations in growth indices and definitions of growth failure

We identified many potential reasons for the lack of generalizability of growth-discriminatory microbiota features, including differences in study populations (Table 1), timing of fecal sample collection, timing of neonatal growth measurements, and statistical techniques used to determine associations (Table 2). Most notably, there was little consensus regarding how growth and growth failure were defined. Growth was assessed using combinations of infant weight, length, and head circumference (HC), and these indices were reported as absolute values, percentiles, rates of change (velocity), or z-scores. Infant age was reported as postnatal days or weeks of life, or as post-menstrual age (PMA) or corrected gestational age (CGA). In some cases, microbiota features were regressed linearly against growth over time, and in other cases they were correlated with a single anthropometric measurement at 36 weeks PMA. Some studies assessed appropriate growth versus growth failure as a binary outcome. Among these studies, growth failure was variably defined as weight < 3^rd^ or < 10^th^ percentile at 36 weeks PMA, as negative change in weight z-score from birth to 36 weeks PMA, or as weekly weight gain rate < 6^th^ percentile – all using a variety of published growth charts (Table 2). Thus, the published literature contains a striking variety of definitions of appropriate neonatal growth and postnatal growth failure.

### Secondary analysis reveals how growth definitions influence taxonomic associations

**Figure 4.**
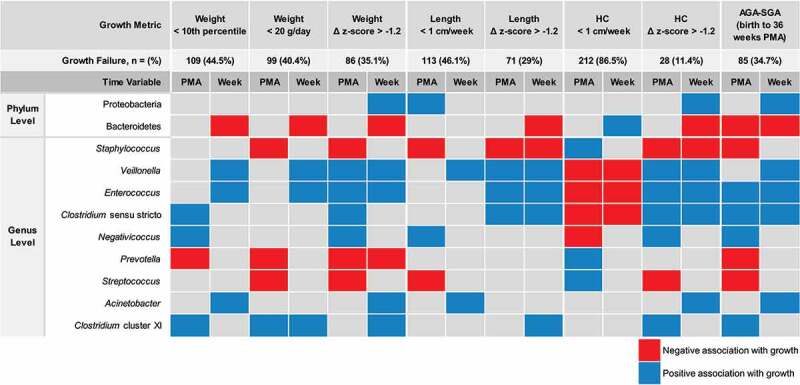
Phylum and genus level associations with postnatal growth based on 16 clinically relevant growth indices. in an exploratory secondary analysis of previously published microbiota sequencing from 245 longitudinally-sampled preterm infant stools, we sought to determine how changing the definition of neonatal growth might change the significantly associated microbes. We tested associations between relative abundance change over time and the binary outcome “appropriate growth” versus “growth failure” using eight clinically relevant definitions and analyzed each longitudinally according to PMA quartiles or postnatal week of life. Significant positive (blue) or negative (red) associations between taxa and postnatal growth are illustrated. *N* = (%) shows that the number of samples from infants classified as having growth failure changes dramatically with each of these 16 definitions. AGA-SGA: change in status from appropriate for gestational age at birth to small for gestational age (weight < 10th percentile) at 36 weeks PMA; HC: head circumference; PMA: post-menstrual age; Week: postnatal week of life.

To gain insight into how different definitions of postnatal growth can impact growth-associating microbiota signatures, we performed an exploratory secondary analysis of a large, previously published data set of preterm infants born < 32 weeks gestational age (GA) and < 1500 g between 2015 and 2016.^31^ This data set included 16S sequencing of 245 fecal samples collected longitudinally throughout the newborn hospitalization. The primary aim of the published study was to determine how the neonatal diet influenced gut microbiota community composition; these microbiota data had not been analyzed with respect to neonatal growth. For our reanalysis, we selected eight definitions of growth failure based on Fenton growth metrics that are both clinically relevant and reported in the literature (all growth velocities and changes were computed from birth to 36 weeks PMA): weight < 10^th^ percentile at 36 weeks PMA; weight gain < 20 g/day; change in weight z-score > −1.2; length velocity < 1 cm/week; change in length z-score > −1.2; HC velocity < 1 cm/week; change in HC z-score > −1.2; and change in status from appropriate for gestational age (AGA) at birth to small for gestational age (SGA; defined as weight < 10^th^ percentile^32^) at 36 weeks PMA.^32–34^ These eight growth failure definitions were longitudinally linked to microbial signatures in two different ways – based on the neonates’ PMA and based on the neonates’ postnatal age (weeks of life). Thus, 16 different definitions of growth were explored. A heat map illustrates microbial taxa that were significantly associated with either appropriate neonatal growth or with growth failure according to these 16 definitions (Figure 4).

Our exploratory secondary analysis of this data set revealed that changing the definition of growth failure influenced which microbial taxa positively or negatively correlated with growth. Within our data set, not one taxonomic feature consistently associated with growth across all definitions. In part, this may be due to the different definitions of growth failure identifying different subsets of neonates. For example, when growth failure was defined as HC velocity < 1 cm/week, the majority of stool samples in this cohort (87%) were from infants classified as having growth failure. However, a minority (11%) of the same samples were from infants classified as having growth failure when the definition of change in HC z-score > −1.2 from birth to 36 weeks PMA was used.

At the phylum level, the relative abundance of Proteobacteria positively associated with neonatal growth with 4/16 definitions, and the relative abundance of Bacteroidetes negatively associated with growth with 7/16 definitions; this latter association was less frequently observed when age was reported as PMA (1/8) rather than as postnatal weeks (6/8). Most definitions negatively associated three genera with growth: *Staphylococcus*, *Prevotella*, and *Streptococcus*. Conversely, multiple definitions positively associated growth with six genera: *Veillonella*, *Enterococcus*, *Clostridium* sensu stricto, *Negativicoccus*, *Acinetobacter*, and *Clostridium* cluster XI. Importantly, whether these taxa significantly associated with growth depended on the choice of PMA versus postnatal weeks as the measure of time. Unexpectedly, defining growth failure as HC velocity < 1 cm/week, which classified more neonates as having growth failure than any other metric, changed the directionality of the association for the majority of taxonomic features. Sub-analyses of neonates fed a majority of mother’s own milk or a majority of donor human milk revealed similar results, although fewer total taxa were significantly associated with growth among these smaller subgroups (Supplementary Figure S1). Taken together, these data indicate that significant associations between microbiota composition and neonatal growth are highly dependent on how both growth and age are defined.

## Discussion

Despite numerous landmark preclinical studies that elucidate mechanisms by which gut microbes regulate postnatal growth, there is a lack of clinically validated biomarkers to predict which preterm newborns are at risk of growth failure, and a lack of microbiota-targeting therapies to prevent and treat this problematic condition. Our systematic review identified 14 studies that have reported relationships between neonatal gut microbiota signatures and postnatal growth. Although every study found significant associations, our qualitative synthesis of this data did not identify any microbiota features that correlated with growth consistently across all (or even most) studies. Each study was designed uniquely, assessing different study populations, different ages at sample collection, different definitions of growth, different sequencing techniques, and different statistical approaches to identify significant microbiota associations. The wide variety of reported definitions of postnatal growth was especially surprising, and our exploratory secondary analysis of a previously published data set illustrates how changing the definition of growth failure dramatically influences which gut microbial taxa positively or negatively correlate with growth.

Our systematic review identified increased abundance of *Staphylococcus* as the most commonly reported microbiota feature in premature infants with growth failure. In full-term infants, *Staphylococcus* is highly abundant in stool from newborns delivered via cesarean section compared to those born vaginally.^35^ Our results are in accord with a study of full-term infants that negatively associates *Staphylococcus* with body mass index at 3 and 52 weeks of age.^36^ However, another study reports a positive association between *Staphylococcus* and weight gain at 1 month of life among vaginally (but not cesarean) delivered full-term infants.^37^ Our review further identified *Klebsiella* as negatively associated with growth; increased abundance of this genus in preterm infants is associated with sepsis and necrotizing enterocolitis.^38^ However, *Klebsiella* is a diverse genus that is commonly found in preterm infant stool. Very few phenotypic or genomic differences have been identified in *Klebsiella* strains isolated from sick infants relative to healthy infants.^39^

We also identified several microbial taxa that multiple studies found to positively correlate with neonatal growth. Many of these taxa, including *Bifidobacterium*, *Bacteroides*, *Clostridium*, *Escherichia*, *Lactobacillus*, *Streptococcus*, and *Veillonella*, are abundant in the stool of healthy, full-term newborns.^40,41^ Among the most dominant microbial community members of the full-term infant gut are Actinobacteria (particularly Bifidobacteriaceae or *Bifidobacterium*);^36,37,42,43^ however, we identified only two studies that found a positive association between *Bifidobacterium* and postnatal growth. Our finding could indicate that a consistent link between *Bifidobacterium* and postnatal growth does not exist. Alternatively, this could reflect the very different, immature gut physiology of preterm relative to full-term neonates; the delayed administration of human milk to preterm neonates, a scenario that provides less of a competitive advantage to HMO-consuming bifidobacteria; or the abnormal initial colonization of premature newborns by microbes native to the neonatal intensive care unit environment.^44^ Consequently, microbial associations with preterm growth should be contextualized to factors that impact the development of the preterm infant gut microbiome.

Management of preterm infants in the neonatal intensive care unit is challenging, and multiple anthropometric indices are monitored to ensure that postnatal growth is sufficient. Most commonly, growth failure is characterized as growth < 10^th^ percentile at 36 weeks PMA. Fenton et al. recently outlined reasons why this definition alone is inadequate.^45^ Specifically, this definition of growth failure has not been shown to predict adverse outcomes, is based only on body weight irrespective of proportional head and length growth and genetic potential, does not account for postnatal weight loss, and is based on an arbitrary growth percentile cutoff. Clinicians routinely monitor body weight, length, and head circumference over time to ensure adequate trajectories of each and to inform targeted and timely nutritional interventions. As a result, studies of preterm neonates contain complex sets of metadata that typically include dozens of growth measurements recorded longitudinally throughout the newborn hospitalization – which lasts a minimum of 3–4 months for the most premature newborns.

Combining longitudinal microbiota sampling with longitudinal growth data presents investigators with a nearly unlimited number of testable associations between gut microbiota features and specific aspects of growth, thus increasing the risk of type I error. Although most software packages contain algorithms to adjust for the possibility of false positives due to testing multiple microbiota features (e.g., false discovery rate), adjustments might not be applied when microbiota data are evaluated repeatedly against multiple definitions of growth or postnatal age. Within our own secondary data analysis, it would be tempting to report microbiota associations by defining growth failure as change in HC z-score > −1.2 and time as PMA (8 significant taxonomic associations), rather than growth failure as length velocity < 1 cm/week and time as postnatal weeks of life (just 2 significant taxonomic associations). Similarly, investigators may account for potentially confounding factors that impact postnatal growth (e.g., co-morbidities including sepsis and necrotizing enterocolitis) in a number of different ways. These factors have been incorporated into statistical models, examined in sub-group analyses, evaluated in metadata tables, built into inclusion and exclusion criteria, or simply ignored.

To advance our understanding of relationships between the neonatal gut microbiome and postnatal growth, we offer five recommendations (Table 3). First, protocols of all neonatal microbiome studies, including interventional and observational studies, should be preregistered. The published record should include a thorough description of all statistical techniques that will be used to analyze the complex and typically longitudinal data sets. Second, given the growing number of microbiota-associated variables known to confound comparisons between cases and controls,^46^ careful attention must be paid to prenatal and perinatal factors that might explain or confound microbiota associations. A rich metadata set should present factors such as mode of delivery, gestational age at birth, sex, antibiotics and other medications, diet, and co-morbidities. Third, if the metadata set includes multiple growth metrics, all tests of association performed by the investigators should be presented in supplemental material. This will help readers understand how robustly specific microbiome features associate with postnatal growth across multiple indices, lend insight into whether certain microbiome features might associate more strongly with weight versus length versus head development, and provide greater transparency regarding the potential risk of type I error. Fourth, future studies should consider alternative approaches to 16S sequencing (e.g., combining whole metagenomic sequencing with metabolomics) to generate mechanistic insights into specific strains of microbes, genes, or metabolic pathways that underlie the reported associations. Recently developed statistical approaches may be used to integrate multi-omic data sets with clinical metadata;^47,48^ these methods should be documented in the preregistered protocol. This information will generate new mechanistic hypotheses that may be tested in preclinical models. Fifth, the published report should include concise, complete, and organized reporting of all laboratory and bioinformatics elements that are essential to the interpretation and comparative analysis of human microbiome studies. One recommended guideline, the Strengthening The Organization and Reporting of Microbiome Studies (STORMS) checklist,^49^ contains an editable table that can be included with supplemental files. Adherence to these recommendations may accelerate the development of new therapies that confer growth-promoting potential to reduce the burden of growth failure among premature neonates.

**Table 3.** Recommendations to improve future studies examining associations between the neonatal gut microbiome and postnatal growth.

1	Preregister all study protocols including proposed statistical techniques.
2	Capture comprehensive metadata including potentially confounding prenatal and perinatal factors.
3	Present all tested associations using any growth definition or index in supplemental material.
4	Consider alternatives to 16S sequencing (e.g., whole metagenomic sequencing with metabolomics).
5	Use microbiome-specific reporting guidelines to concisely and completely report essential laboratory and statistical details.

## Methods

This systematic review was registered in PROSPERO [CRD42022361402] and was conducted according to the guidelines outlined in Preferred Reporting Items for Systematic Reviews and Meta-Analyses (PRISMA).^50^

### Study identification strategy

Four databases were searched (PubMed, Web of Science, Cochrane Library, and Medline/Ovid) in September 2022. Studies relevant to gut microbiome associations with preterm infant growth were identified using the following search terms: (“infant,” OR “neonate,” AND “premature,” OR “preterm”), OR “preterm infant,” OR “premature infant,” OR “preterm neonate,” OR “premature neonate,” AND “microbiome,” OR “microbiota,” OR “flora,” OR “gut microbiome,” OR “gut microbiota,” OR “gut microbes,” OR “gut flora,” OR “gastrointestinal microbiome,” OR “gastrointestinal microbes,” OR “gastrointestinal microbiota,” OR “gastrointestinal flora,” OR “intestinal microbiome,” OR “intestinal microbiota,” OR “intestinal microbes,” OR “intestinal flora,” OR “fecal microbiome,” OR “fecal microbiota,” OR “fecal microbes,” AND “growth,” OR “growth failure,” OR “extrauterine growth restriction,” OR “growth faltering,” OR “postnatal growth,” OR “weight,” OR “height,” OR “length,” OR “head circumference,” OR “z-score,” OR “percentile.” Only studies published in the English language were included. Additional records were identified in PubMed searches.

### Inclusion and exclusion criteria

Studies were screened according to the following inclusion criteria: (1) study design of prospective, retrospective, longitudinal, cross-sectional, case-control, or randomized controlled trial; (2) studied preterm infants during their newborn hospital admission; (3) evaluated gut microbiome composition; (4) evaluated postnatal growth; and (5) assessed microbiota in relation to growth. Studies were excluded if they met the following criteria: (1) systematic review, narrative review, opinion, perspective, commentary, meta-analysis, animal study, or *in vitro* study; (2) studies on non-preterm infants; (3) protocol studies, conference abstracts, or studies not yet completed; (4) studies published in a language other than English; and (5) studies that lacked analyses relevant to associations between gut microbiome composition and postnatal growth.

### Study selection and data synthesis

Studies identified in the database search were exported and screened using a reference manager (Zotero) based on titles and abstracts. Full-text screens were performed when necessary to determine eligibility. Eligible studies were assessed and the following data were extracted: author, year of publication, geographical location, study type, sample size, gestational age at birth, birth weight, neonatal diet, sample collection methods, microbiome analysis (including DNA extraction) method, and therapeutic interventions when applicable. For each study, we collected data reporting associations of preterm postnatal growth with gut microbiome composition including microbiota alpha diversity, beta diversity, and relative abundance of specific taxa.

Because the variability of study methods and outcomes precluded a quantitative data synthesis, we qualitatively depicted microbial taxa that were reported to have positive or negative associations with early postnatal growth in a series of Venn diagrams. These taxa include a range of classifications from phylum to genus, as reported in the individual studies. When individual studies reported multiple statistical associations within the same data set, only associations that remained significant after adjusting for confounding factors and false discovery rate are included in our qualitative synthesis. A taxon was excluded from our qualitative synthesis if the same study reported both positive and negative associations between that taxon and growth. Venn diagrams were created with the BioRender.com platform.

### Secondary microbiota analysis

The secondary analysis in this report was generated from the Ford et al. study,^31^ a prospective cohort study (NCT02573779) conducted at Texas Children’s Hospital – Pavilion for Women and approved by the Institutional Review Board at Baylor College of Medicine. This study enrolled 125 very low birth weight preterm infants < 32 weeks gestational age. Stool samples, including the first meconium sample, were collected every week during the first 6 weeks of life. Inclusion/exclusion criteria, baseline demographics, and clinical outcomes are described in the previous report. In all, 249 stool samples were included in the secondary analysis. Associations between the gut microbiota and neonatal growth had not been investigated in the published study.

For our secondary analysis, we incorporated anthropometric indices that classify infants as having “growth failure” or “appropriate growth.” Growth failure definitions include: weight at 36 weeks PMA < 10^th^ percentile using Fenton 2013 growth curves;^33^ weight velocity from birth to 36 weeks PMA < 20 g/day; length and HC velocities from birth to 36 weeks PMA < 1 cm/week; change in weight, length, or HC z-score > −1.2 from birth to 36 weeks PMA;^34^ and infants who were born AGA then became SGA (weight < 10^th^ percentile at 36 weeks PMA^32^). Infants who were not classified as growth failure were classified as having appropriate growth. In the original prospective study, two infants died before 36 weeks PMA; therefore, their stools were excluded from the secondary analysis, resulting in a total of 245 stool samples analyzed in this report.

Raw sequences of the V4 region of the 16S rRNA gene were generated and processed as previously described.^31^ Analyses presented in this review were performed with ATIMA (Agile Toolkit for Incisive Microbial Analysis), a microbiome visualization tool developed by the Alkek Center for Metagenomics and Microbiome Research (CMMR) at Baylor College of Medicine. Data were rarified to 1015 reads per sample as done in the source study. For each definition of growth and neonatal age, all stools were categorized using the binary outcome growth failure versus appropriate growth, and taxonomic trends over time were analyzed either by week of life or by post-menstrual age (PMA). Statistical significance was determined by the Kruskal-Wallis test adjusted for false discovery rate. The taxonomic mean abundance was set at a threshold of ≥ 0.05%, unclassified taxa were excluded, the top 4 phyla and top 9 genera were ranked by the significance of the p-value, and these data were extracted. Taxa with relative abundances that changed significantly over time in one binary growth outcome but not the other were depicted in a heat map constructed in Microsoft Excel. Taxa with increasing abundance in growth failure or decreasing abundance in appropriate growth were designated as negatively associated with growth, whereas taxa with increasing abundance in appropriate growth or decreasing abundance in growth failure group were designated as positively associated with growth.

## Supplementary Material

Supplemental Material

## Data Availability

All data and concepts described arise from the analysis of the literature detailed above (DOI: 10.1093/ajcn/nqz006).
