# Supplementary material for: A systematic review of associations between gut microbiota composition and growth failure in preterm neonates
Source: Gut Microbes. 2023 Mar 16;15(1):2190301. doi: 10.1080/19490976.2023.2190301 (PMC10026866; doi:10.1080/19490976.2023.2190301)
Supplement: Supplemental Material [file KGMI_A_2190301_SM4781.pdf]

Supplementary Figure 1

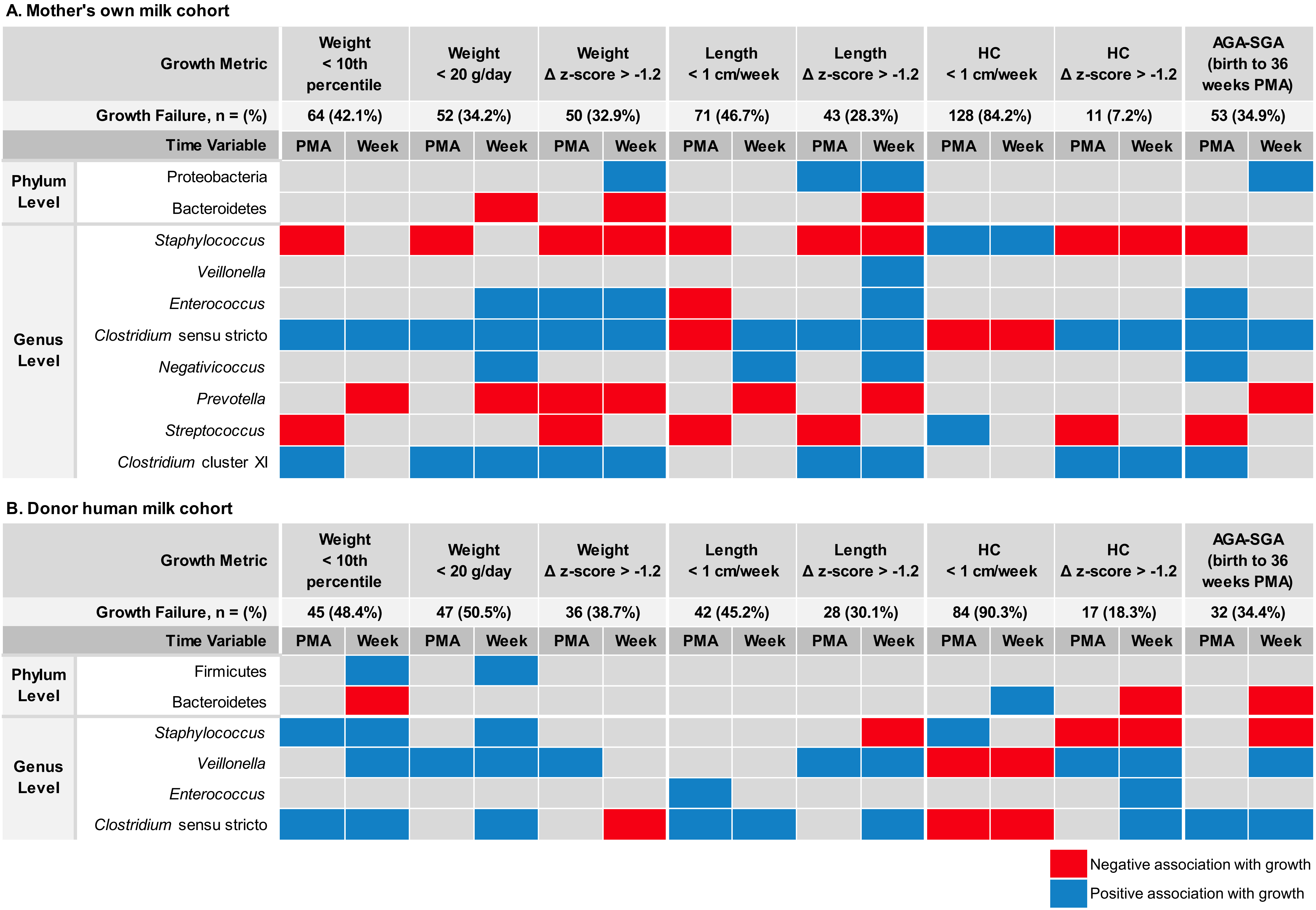

Supplementary Figure 1. Phylum and genus level associations with postnatal growth sub-analyzed by neonatal diet. Significant positive (blue) or negative (red) associations between taxa and postnatal growth are illustrated. Associations were determined among neonates fed (A) a majority of mother's own milk or (B) a majority of donor human milk based on 16 clinically relevant growth indices. N = (%) shows that the number of samples from infants classified as having growth failure changes dramatically with each of these 16 definitions. AGA-SGA, change in status from appropriate for gestational age at birth to small for gestational age (weight < 10th percentile) at 36 weeks PMA; HC, head circumference; PMA, post-menstrual age; Week, postnatal week of life.
